# Relationship of Cisplatin-Related Adverse Health Outcomes With Disability and Unemployment Among Testicular Cancer Survivors

**DOI:** 10.1093/jncics/pkaa022

**Published:** 2020-03-20

**Authors:** Sarah L Kerns, Chunkit Fung, Sophie D Fossa, Paul C Dinh, Patrick Monahan, Howard D Sesso, Robert D Frisina, Darren R Feldman, Robert J Hamilton, David Vaughn, Neil Martin, Robert Huddart, Christian Kollmannsberger, Deepak Sahasrabudhe, Shirin Ardeshir-Rouhani-Fard, Lawrence Einhorn, Lois B Travis

**Affiliations:** p1 University of Rochester Medical Center, Rochester, NY, USA; p2 Oslo University Hospital, Oslo, Norway; p3 Indiana University, Indianapolis, IN, USA; p4 Brigham and Women’s Hospital, Boston, MA, USA; p5 University of South Florida, Tampa, FL, USA; p6 Memorial Sloan Kettering Cancer Center, New York, NY, USA; p7 Princess Margaret Cancer Center, Toronto, Canada; p8 University of Pennsylvania, Philadelphia, PA, USA; p9 Dana-Farber Cancer Institute, Boston, MA, USA; p10 Royal Marsden Hospital, London, UK; p11 University of British Columbia, Vancouver, British Columbia, Canada

## Abstract

**Background:**

Few data exist on the relationship of cisplatin-related adverse health outcomes (AHOs) with disability, unemployment, and self-reported health (SRH) among testicular cancer survivors (TCS).

**Methods:**

A total of 1815 TCS at least 1 year postchemotherapy underwent clinical examination and completed questionnaires. Treatment data were abstracted from medical records. A cumulative burden of morbidity score (CBM_Pt_) encompassed the number and severity of platinum-related AHOs (peripheral sensory neuropathy [PSN], hearing loss, tinnitus, renal disease). Multivariable regression assessed the association of AHOs and CBM_Pt_ with employment status and SRH, adjusting for sociodemographic and clinical characteristics. Unemployment was compared with a male normative population of similar age, race, and ethnicity.

**Results:**

Almost 1 in 10 TCS was out of work (2.4%, disability leave; 6.8%, unemployed) at a median age of 37 years (median follow-up = 4 years). PSN (odds ratio [OR] = 2.89, 95% confidence interval [CI] = 1.01 to 8.26, grade 3 vs 0, *P* = .048), renal dysfunction defined by estimated glomerular filtration rate (OR = 12.1, 95% CI = 2.06 to 70.8, grade 2 vs 0, *P* = .01), pain (OR = 10.6, 95% CI = 4.40 to 25.40, grade 2 or 3 vs 0, *P* < .001), and CBM_Pt_ (OR = 1.46, 95% CI = 1.03 to 2.08, *P* = .03) were associated with disability leave; pain strongly correlated with PSN (*r*^2^ = 0.40, *P* < .001). Statistically significantly higher percentages of TCS were unemployed vs population norms (age-adjusted OR = 2.67, 95% CI = 2.49 to 3.02, *P* < .001). PSN (OR = 2.44, 95% CI = 1.28 to 4.62, grade 3 vs 0, *P* = .006), patient-reported hearing loss (OR = 1.82, 95% CI = 1.04 to 3.17, grade 2 or 3 vs 0, *P* = .04), and pain (OR = 3.75, 95% CI = 2.06 to 6.81, grade 2 or 3 vs 0, *P* < .001) were associated with unemployment. Increasing severity of most cisplatin-related AHOs and pain were associated with statistically significantly worse SRH.

**Conclusions:**

Our findings have important implications regarding treatment-associated productivity losses and socioeconomic costs in this young population. Survivorship care strategies should include inquiries about disability and unemployment status, with efforts made to assist affected TCS in returning to the workforce.

Testicular cancer (TC) is the most common cancer among men aged 18–39 years (1). With effective cisplatin-based chemotherapy (2), overall 5-year relative survival rates exceed 95% (3). Because most patients are either in or just entering the workforce when diagnosed, an understanding of factors that may influence their ability to engage in employment after treatment is crucial, both in terms of individual rehabilitation and societal costs. Cancer survivors in general are more likely to suffer from impaired health, leading to a loss of workability compared with healthy individuals (4,5). In childhood cancer survivors, severe and life-threatening conditions statistically significantly increase the likelihood of receiving social security disability insurance and supplemental security income compared with survivors with mild to moderate or no adverse health conditions (6).

Data on employment status among TC survivors (TCS), however, are sparse and derive largely from Europe (5,7–10). Swedish TCS (8) given over 4 cisplatin-based chemotherapy courses had fivefold risks of disability pension, characterized by at least 1 year of work leave for illness. Survivors also reported a higher average number of annual days of work loss for up to 10 years after diagnosis, but platinum-related toxicities were not considered. Other European TCS studies found no statistically significant increased risk of work loss but were limited in size (range = 71–206) (5,7,9,10). Cisplatin-treated TCS may experience statistically significant adverse events, including hearing loss, tinnitus, and peripheral neuropathy (1,11), with 40% reporting at least 3 adverse health outcomes (AHOs) after a median follow-up of 4 years (1). It remains unclear, however, to what extent AHOs might either influence employment outcomes, such as disability leave, or affect self-reported health (SRH) status, which itself is associated with employment status (12).

To provide new information regarding employment outcomes in a largely US-based cohort of TCS, we examined these endpoints in relation to platinum-related AHOs and sociodemographic and clinical features among 1815 TCS enrolled in a large, multicenter clinical investigation (1,11). We also evaluated the association of platinum-related AHOs with SRH. We hypothesized that cisplatin-related AHOs are associated with disability and unemployment.

## Methods

### Study Population

The Platinum Study was approved by institutional review boards at all sites, and each participant provided informed consent. Cohort methods were previously described (see Supplementary Materials, available online) (1,13). In brief, eligible participants had histological or serological diagnosis of germ cell tumor at 60 years of age or younger, finished all cisplatin-based chemotherapy over or equal to 1 year preenrollment, and were disease free at study entry; thus, no participant was undergoing chemotherapy for cancer recurrence. Participants completed comprehensive questionnaires, reported prescription medication use with indication, and underwent physical examinations and audiometric assessment, described previously (1,13). Cancer diagnosis and treatment data were abstracted from medical records. Because 90% of germ cell tumors were gonadal, for simplicity, all participants are referred to as TCS.

### Sociodemographic Characteristics, Health Behaviors, and AHOs

Sociodemographic characteristics (including employment), health behaviors, and AHOs were assessed using validated patient-reported outcome (PRO) questionnaires (see Supplementary Materials, available online). These included the European Organisation for Research and Treatment of Cancer Chemotherapy-Induced Peripheral Neuropathy (EORTC-CIPN-20) (14) instrument and the Scale for Chemotherapy-Induced Neurotoxicity (15). In a subset of participants, selected objective assessments, including extensive audiometry and serum creatinine measurements, were undertaken. Presence or history of AHOs was queried at clinical assessment, and participants reported current prescription medication use with indication. Participant responses to PROs were mapped to individual AHOs, with severity graded (0–4) using a modified version of the CTCAE-4.03 (16), as previously published (11) (Supplementary Table 1, available online). Audiometry was done as described previously (13,17) and categorized according to American Speech Language Hearing Association criteria (17). The estimated glomerular filtration rate (eGFR) (18) evaluated renal function (see Supplementary Materials, available online).

The cumulative burden of morbidity platinum (CBM_Pt_) scores (Supplementary Table 2, available online) was calculated based on the number and severity of AHOs previously related to cisplatin, that is, peripheral sensory neuropathy (PSN), hearing loss, tinnitus, and renal disease (19,20), following methods adapted from Geenen et al. (21) as described previously (11).

Employment status was determined at clinical evaluation: “What is your current employment status?” Responses included unemployed, part-time employment, full-time employment, retired, disability leave, and prefer not to say. Unemployment rates by age group between TCS and the general US population, matching on race or ethnicity, were ascertained from the 2016 Behavioral Risk Factor Surveillance System (BRFSS) (22). BRFSS is a cross-sectional, random digit–dialed telephone survey of more than 400 000 US adults aged 18 years or older, using standard modules. Employment questions are similar to our survey (see Appendix and http://www.cdc.gov/brfss/index.htm) and have been used to characterize population employment status in a broad spectrum of studies (23–34). Differences in unemployment between TCS and BRFSS (restricted to non-Hispanic white males without a cancer diagnosis) by age category were assessed using chi-square tests. A Cochran Mantel-Haenszel test assessed the overall age-adjusted difference. SRH was evaluated with the validated question (35): “In general, would you say your health is excellent, very good, good, fair, or poor?”

### Statistical Methods

Discrete and continuous data were described using numbers (percentages) and medians (ranges). Clinical or sociodemographic characteristics, health behaviors, individual AHOs, and the CBM_Pt_ score were each tested for association with employment status using Pearson chi-square test; if 20% or more of cells within a contingency table had expected counts less than five, Fisher’s exact test was used.

Multivariable logistic regression models were developed using backward selection to identify clinical and sociodemographic variables associated with employment status, with *P* less than .05 defining variables for final inclusion. Individual AHOs were then assessed for association with disability leave or unemployment in separate models adjusted for these covariates. Time since chemotherapy completion was added into models as a covariate, and interaction with each AHO was assessed with *P* less than .05 indicating statistical significance.

Multivariable ordinal logistic regression models assessed association between different grades of each AHO and SRH, adjusting for previously identified SRH-related covariates in the general population (36–40): age, race, educational level, physical activity, employment, and smoking status. Assumptions of proportionality of odds across response categories in ordinal logistic regression models were confirmed by comparing the Bayes information criterion for the proportional odds model to the Bayesian Information Criterion from a partial proportional odds model.

All analyses were performed with Stata v14.1 (41) except for SAS-SURVEYFREQ, with the Rao-Scott chi-squarecomparing our population vs BRFSS while accounting for the complex sample design; published BRFSS sampling weights were used, with weight = 1 applied for our population.

All tests were two-sided, and a *P* value of less than .05 was considered statistically significant.

## Results

The median age at evaluation for 1815 TCS was 37 years (range = 18–75 years) and median time since chemotherapy completion was 3.8 years (range = 1–35 years) (Table 1). Most participants received standard chemotherapy regimens: 3 cycles of bleomycin, etoposide, and cisplatin (BEPX3; N = 644, 35.5%), EPX4 (N = 540, 29.8%), or BEPX4 (N = 308, 17.0%), with 17.7% receiving other regimens. The population was largely white (87.7%), college educated (64.1%), and employed full-time (81.1%). About 2.4% of TCS were on disability leave and 6.8% were unemployed.

**Table 1.** Clinical and sociodemographic characteristics and health behaviors of 1815 survivors of cisplatin-treated TC

Characteristics	No. (%)
Total	1815
Clinical characteristics	
Age at diagnosis, y	
Median [range]	30 [10–60]
<20	140 (7.7)
20–29	734 (40.4)
30–39	583 (32.1)
40–49	287 (15.8)
50–60	71 (3.9)
Age at clinical evaluation, y	
Median [range]	37 [18–75]
<20	17 (0.9)
20–29	414 (22.8)
30–39	650 (35.8)
40–49	433 (23.9)
50–59	241 (13.3)
≥60	60 (3.3)
Histologic type	
Seminoma	447 (24.6)
Nonseminoma or mixed germ cell tumor	1328 (73.2)
Germ cell tumor, not otherwise specified	40 (2.2)
Site*	
Testis	1597 (89.0)
Extragonadal	197 (11.0)
Chemotherapy regimen	
BEPX3	644 (35.5)
EPX4	540 (29.8)
BEPX4	308 (17.0)
VIPX4/VIPX5	46 (2.5)
Cisplatin-based chemotherapy ≥5 cycles^†^	78 (4.3)
Other cisplatin-based chemotherapy^‡^	199 (10.9)
Cumulative dose of cisplatin, mg/m^2^	
Median [range]^§^	400 [100–1403]
<300	129 (7.2)
300	644 (35.8)
301–399	79 (4.4)
400	840 (46.6)
>400	109 (6.1)
Cumulative dose of bleomycin, IU	
Median [range]	270 [11–630]
0	784 (43.2)
>0–180 000	100 (5.5)
181 000–270 000	684 (37.7)
271 000–360 000	235 (12.9)
>360 000	12 (0.7)
Calendar year of chemotherapy completion^‖^	
Before 2000	173 (9.6)
2000–2009	566 (31.4)
2010–2018	1061 (58.9)
Retroperitoneal lymph node dissection^¶^	
Yes	836 (46.5)
No	962 (53.5)
Time from chemotherapy completion to clinical evaluation, y^#^	
Median [range]	3.8 [1–35.2]
<2	556 (31.8)
2–5	560 (32.0)
6–9	264 (15.1)
10–14	195 (11.2)
15–19	103 (5.9)
≥20	71 (4.1)
Sociodemographic characteristic	
Race**	
White	1495 (87.7)
African American	18 (1.1)
Asian	79 (4.6)
Other	113 (6.6)
Marital status^††^	
Single or never married	574 (33.2)
Married or living as married	1045 (60.5)
Divorced or separated	109 (6.3)
Education^‡‡^	
High school or less	207 (11.9)
After high school but not college graduate	417 (24.0)
College or university graduate	729 (41.9)
Postgraduate level	386 (22.2)
Employment status^§§^	
Employed full-time	1401 (81.1)
Employed part-time	134 (7.8)
Unemployed	117 (6.8)
Retired	34 (2.0)
On disability leave	41 (2.4)
Health behavior	
Smoking status^‖‖^	
Never	1001 (57.2)
Former	594 (34.0)
Current	154 (8.8)
Average number of alcoholic drinks in past year^¶¶^	
Rarely or never	360 (20.6)
≤4/wk	748 (42.9)
5/wk to 1/d	412 (23.6)
≥2 daily	225 (12.9)
Engage in vigorous physical activity (≥6 METs)^##^	
Yes	1178 (65.1)
No	633 (34.9)

* Germ cell tumor site was not available for 21 participants. BEPX3 = 3 cycles of bleomycin, etoposide, and cisplatin; BEPX4 = 4 cycles of bleomycin, etoposide and cisplatin; EP4 = 4 cycles of etoposide and cisplatin; IU = international units; METs = metabolic equivalents; TC = testicular cancer; VIPX4 = 4 cycles of etoposide, ifosfamide, and cisplatin; VIPX5 = 5 cycles of etoposide, ifosfamide, and cisplatin.

† Includes 5 cycles (n = 29), 6 cycles (n = 38), 7 cycles (n = 5), and 8 or more cycles (n = 6) of cisplatin-based chemotherapy. Ten patients were treated for a recurrence, with 1, 3, 4, and 2 receiving a total of 6, 7, 8, and more than 8 cycles of chemotherapy, respectively. All chemotherapy was completed at least 1 year before study entry (see "Methods").

‡ In addition to patients given regimens that are itemized in this table, the total number also includes those who received EPX2 (n = 27), BEPX2 (n = 27), EPX3 (n = 62), VIPX3 (n = 6), three cycles of other cisplatin-based chemotherapy (n = 3), 4 cycles of other cisplatin-based chemotherapy (n = 44), 4 cycles of cisplatin plus ifosfamide (n = 3), cisplatin, bleomycin, and vinblastine (n = 5), and cisplatin-based chemotherapy with no further details (n = 22).

§ Cisplatin dose information was not available for 14 participants.

‖ Chemotherapy completion date was not available for 15 participants.

¶ Retroperitoneal lymph node dissection information was not available for 17 participants.

# Information on time since completion of chemotherapy was not available for 66 participants.

** Race not stated for 110 participants.

†† Marital status not stated for 87 participants.

‡‡ Educational status not stated for 76 participants.

§§ Employment status not stated for 88 participants.

‖| Smoking status not stated for 66 participants.

¶¶ Alcohol use not stated for 70 participants.

## Physical activity information was not provided by 4 participants. Nine activities were surveyed, some of which were vigorous-intensity activities, such as 30 minutes of running per week (55–57).

**Figure 1.**
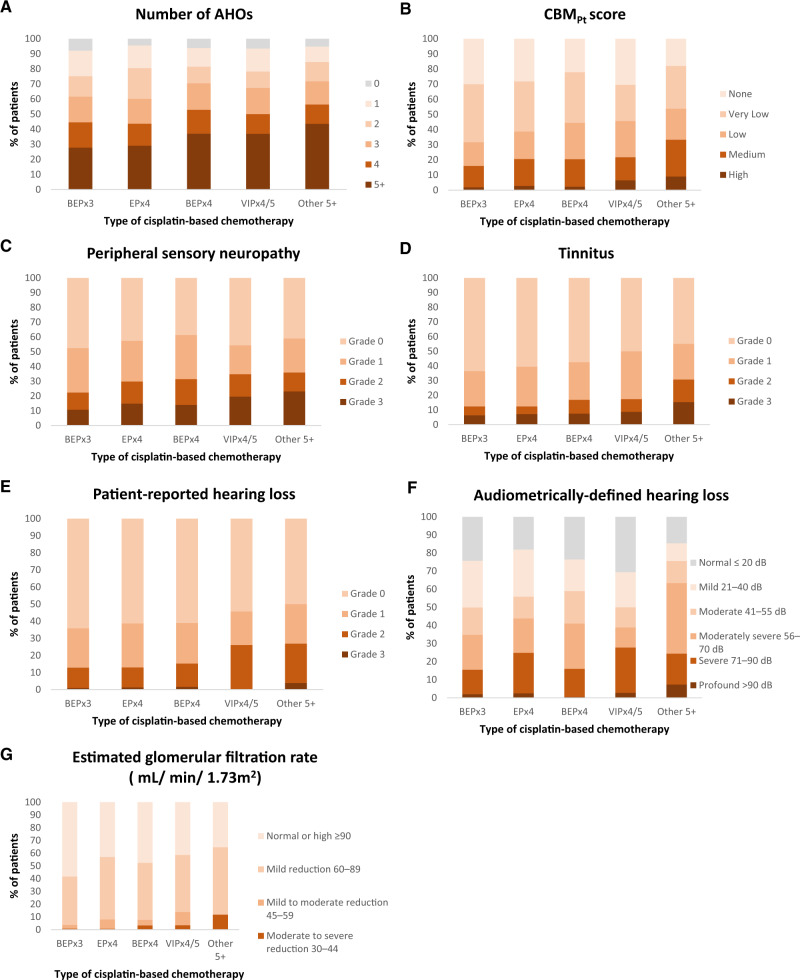
Prevalence of various adverse health outcomes (AHOs) by type of cisplatin-based chemotherapy. **A**) Any AHO using PROs, **B**) cumulative burden of morbidity-platinum (CBM_Pt_) score, **C**) peripheral sensory neuropathy (PSN), **D**) tinnitus, **E**) patient-reported hearing loss, **F**) audiometrically defined hearing loss using American Speech-Language-Hearing Association criteria (17), and **G**) estimated glomerular filtration rate (eGFR) (mL/min/1.73 m^2^) (18). The CBM_Pt_ score was calculated based on the number and severity of AHOs previously related to cisplatin exposure: PSN, hearing loss, tinnitus, and renal disease. The eGFR formula (18) includes two additional categories of renal dysfunction (ie, 15–29 mL/min/1.73 m^2^ [severe reduction] and <15 mL/min/1.73 m^2^ [renal failure]); because no study participant had an eGFR value at these levels, these categories are not shown in the figure. BEP = bleomycin, etoposide, and cisplatin; dB = decibel; EP = etoposide and cisplatin; other 5+ = cisplatin-based chemotherapy at least 5 cycles; VIP = etoposide, ifosfamide, and cisplatin.

Table 2 lists the prevalence and severity of individual AHOs, and Figure 1 shows AHOs by chemotherapy regimen. Following BEPX3 and EPX4, at least 3 AHOs were reported by 61.7% and 60.2% of TCS with larger percentages after BEPX4, 4 cycles of etoposide, ifosfamide, and cisplatin (VIPX4)/5 cycles of etoposide, ifosfamide, and cisplatin (VIPX5), or 5+ cycles of other cisplatin-based chemotherapy (70.5%, 67.4%, 71.8%, respectively, *P* = .01) (Figure 1A). Proportions of TCS with medium or high CBM_Pt_ scores varied by chemotherapy and increased with increasing cycle number (16.0%, 20.6%, 20.5%, 21.7%, and 33.3% after BEPX3, EPX4, BEPX4, VIPX4/5, and other 5+ cycles, respectively, *P* = .002; Figure 1B). Percentages of patients with neuropathy, tinnitus, hearing loss (patient reported or audiometrically defined), and reduced eGFR also increased with increasing chemotherapy cycles, as did outcome severity (Figure 1, C–G). For example, proportions of TCS with tinnitus (any grade) were 36.5%, 39.4%, 42.5%, 50.0%, and 55.1%, respectively, after BEPX3, EPX4, BEPX4, VIPX4/5, and other 5+ cycles, respectively, *P* = .009; for grades 2–3, percentages were 12.4%, 12.4%, 16.9%, 17.4%, and 30.8%, respectively (*P* < .001). Supplementary Table 3 (available online) shows numerical representations of Figure 1. Overall, audiometric assessments showed 34.9% TCS with moderate (41–55 dB) to moderately severe (56–70 dB) hearing loss and 19.5% with severe (71–90 dB) to profound (>90 dB) hearing loss. Calculations of eGFR indicated mild-to-moderate or moderate-to-severe reductions in renal function in 6.5% TCS.

**Table 2.** Prevalence of AHOs by severity grade among 1815 cisplatin-treated TCS

		Severity grade, No. (%)					
AHO		All grades		Grade 1	Grade 2	Grade 3	Grade 4
Included in CBM_Pt_ score*							
Patient-reported hearing loss^†, ‡^		688 (37.9)		429 (23.6)	238 (13.1)	24 (1.3)	0
Tinnitus^§^		708 (39.0)		450 (24.8)	127 (7.0)	131 (7.2)	NA
Peripheral sensory neuropathy^‖^		1004 (55.3)		516 (28.4)	249 (13.7)	239 (13.2)	NA
Patient-reported^¶^ renal disease		44 (2.4)		41 (2.3)	3 (0.2)	NA	NA
Other AHOs							
eGFR-defined renal disease^#^		489 (50.1)		423 (43.3)	66 (6.8)	0	NA
Autonomic neuropathy**		483 (26.6)		352 (19.4)	99 (5.5)	32 (1.8)	NA
Raynaud phenomenon^§^		590 (32.5)		262 (14.4)	181 (10.0)	153 (8.4)	NA
Pain^††^		440 (24.2)		244 (13.4)	167 (9.2)	29 (1.6)	NA
Hypercholesterolemia^¶^		123 (6.8)		NA	123 (6.8)	NA	NA
Hypertriglyceridemia^‡‡^		8 (0.4)		NA	8 (0.4)	NA	NA
Hypertension^¶^		154 (8.5)		NA	154 (8.5)	NA	NA
Diabetes^¶^		54 (3.0)		NA	28 (1.5)	26 (1.4)	NA
Coronary artery disease^¶^		45 (2.5)		6 (0.3)	19 (1.1)	20 (1.1)	NA
Transient ischemic attack^§§^		10 (0.6)		10 (0.6)	NA	NA	NA
Stroke^‖‖^		9 (0.5)		NA	9 (0.5)	NA	0
Peripheral artery disease^¶^		72 (4.0)		35 (1.9)	20 (1.1)	17 (0.9)	NA
Thromboembolic event^#^		124 (6.8)		NA	58 (3.2)	66 (3.6)	NA
Obesity^¶¶^		1254 (69.1)		NA	754 (41.5)	437 (24.1)	63 (3.5)
Thyroid disease^¶^		47 (2.6)		25 (1.4)	22 (1.2)	NA	NA
Erectile dysfunction^¶^		489 (26.9)		263 (14.5)	226 (12.5)	NA	NA
Anxiety and/or depression^‡‡^		91 (5.0)		NA	91 (5.0)	NA	NA
Hypogonadism^‡‡^		154 (8.5)		NA	154 (8.5)	NA	NA

* The CBM_Pt_ score consists of patient-reported outcomes for hearing loss, tinnitus, PSN, and kidney disease. AHO = adverse health outcome; CBM_Pt_ = cumulative burden of morbidity-platinum; eGFR = estimated glomerular filtration rate; EORTC-CIPN-20 = European Organisation for Research and Treatment of Cancer Chemotherapy-Induced Peripheral Neuropathy 20-item; NA = not applicable (data needed to assign grade were not captured); PSN = peripheral sensory neuropathy; SCIN = Scale for Chemotherapy-Induced Long-Term Neurotoxicity; TC = testicular cancer; TCS = testicular cancer survivors.

† Assessed using the Hearing Handicap Inventory by Ventry and Weinstein (58) administered at the time of clinical evaluation. For each item, participants were asked to report the age (in years) at first occurrence. If onset of symptoms was before the age at TC diagnosis, those responses were not considered when assigning severity grade.

‡ Audiometrically assessed hearing loss was not graded according to Common Terminology Criteria for Adverse Events v4.03 metrics but was instead graded according to the more granular American Speech-Language-Hearing Association criteria (17), reflecting the detail that was captured.

§ Assessed with the SCIN questionnaire (15).

‖ Assessed with the EORTC-CIPN-20 questionnaire (14), the SCIN questionnaire (15), and patient-reported current prescription medication use. Prescription medications were only considered if the respondent stated that the indication was for neuropathy.

¶ Assessed using patient-reported information on physician-diagnosed condition and current prescription medication use. Prescription medications were only considered if the respondent stated that the indication was for the AHO of interest.

# The eGFR was calculated among 976 TCS following methods in Levey et al. (18). See Supplemental Table A1 (available online) for details.

** Assessed with the EORTC-CIPN-20 questionnaire (14).

†† Assessed with the SF36 questionnaire (35).

‡‡ Assessed using current prescription medication use. Prescription medications were only considered if the respondent stated that the indication was the AHO of interest.

§§ Assessed using patient-reported information on physician-diagnosed condition.

‖‖ Assessed using patient-reported information on physician-diagnosed condition and surgical procedures to address stroke.

¶¶ Defined based on body mass index calculated using height and weight measurements taken at the time of clinical evaluation. The median body mass index was 27 kg/m^2^ (range = 18–67 kg/m^2^) and did not vary by treatment group, as defined in Table 1. Waist circumference (median = 37.0 inches) was routinely measured for each patient; it did not differ by treatment group and was not included in the AHO list or any subsequent analyses.

Table 3 depicts associations of AHOs and clinical and sociodemographic characteristics with employment status. Compared with TCS employed full-time (median age = 37 years), unemployed and part-time employed TCS were slightly younger (median age = 30 and 29 years, respectively), whereas those on disability leave were slightly older (median age = 44 years). Statistically significantly greater proportions of TCS employed full-time were white (90.3%) and highly educated (70.2%= college or postgraduate) than those on disability and leave (79.5% and 26.8%, respectively; *P* < .001 each). The latter were more likely to report prescription antipsychotropic medication use (24.4%) and lack of vigorous physical activity (61.0%) compared with those employed full-time (4.4% and 29.9%, respectively; *P* < .001 each). Greater percentages of TCS on disability leave experienced grade 2 or 3 cisplatin-related AHOs (1 grade 2 or 3 AHO: 2.4%; 2 grade 2 or 3 AHOs: 2.4%; 3 grade 2 or 3 AHOs: 19.5%) compared with TCS employed full-time (3.6%, 5.8%, and 4.7%, respectively; *P* < .001) despite similar times since chemotherapy completion. SRH was markedly worse among TCS on disability leave (53.7% fair or poor) or unemployed (16.2% fair or poor) compared with those employed full-time (4.1% fair or poor; *P* < .001). Table 4 shows the percentages of unemployed TCS by age group vs BRFSS population norms (overall age-adjusted odds ratio [OR] = 2.75, 95% confidence interval [CI] = 2.49 to 3.02, *P* < .001). Supplementary Table 4 (available online) shows these results when the BRFSS sample also includes other cancer survivors, with the results not materially different.

**Table 3.** Characteristics of 1727* survivors of cisplatin-treated TC according to current employment status

	Employed full-time	Employed part-time	Unemployed	Retired	On disability	
Characteristics	(N = 1401)	(N = 134)	(N = 117)	(N = 34)	(N = 41)	*P*
Clinical characteristics						
Age at clinical evaluation, y						
Median [range]	37 [18–70]	29 [18–71]	30 [18–63]	60 [43–75]	44 [23–64]	<.001^†^
<20	3 (0.2)	6 (4.5)	6 (5.1)	0	0	
20–29	272 (19.4)	65 (48.5)	49 (41.9)	0	4 (9.8)	
30–39	534 (38.1)	35 (26.1)	33 (28.2)	0	9 (22.0)	
40–49	368 (26.3)	13 (9.7)	14 (12.0)	6 (17.7)	17 (41.5)	
50–59	196 (14.0)	6 (4.5)	14 (12.0)	11 (32.4)	9 (22.0)	
≥60	28 (2.0)	9 (6.7)	1 (0.9)	17 (50.0)	2 (4.9)	
Time from chemotherapy completion to clinical evaluation, y						
Median (range)	4.2 [1.0–34.9]	2.6 [1.0–30.4]	2.4 [1.0–20.1]	12.1 [1.1–35.2]	4.2 [1.0–23.7]	<.001^†^
<2	417 (30.3)	57 (43.2)	52 (45.2)	4 (13.8)	10 (25.6)	
2–5	428 (31.1)	47 (35.6)	44 (38.3)	4 (13.8)	13 (33.3)	
6–9	222 (16.1)	13 (9.9)	10 (8.7)	3 (10.3)	7 (18.0)	
10–14	162 (11.8)	7 (6.1)	7 (6.5)	6 (20.7)	6 (16.4)	
≥15	149 (10.8)	8 (6.1)	2 (1.7)	12 (41.4)	3 (7.7)	
Chemotherapy regimen						
BEPX3	511 (41.0)	41 (33.3)	42 (37.8)	9 (28.1)	11 (35.5)	.004^†^
EPX4	432 (34.6)	39 (31.7)	29 (26.1)	17 (53.1)	9 (29.0)	
BEPX4	213 (17.1)	30 (24.4)	31 (27.9)	5 (15.6)	8 (25.8)	
VIPX4/VIPX5	33 (2.7)	6 (4.9)	3 (2.7)	0	1 (3.2)	
Other cisplatin-based chemotherapy ≥5 cycles	58 (4.7)	7 (5.7)	6 (5.4)	1 (3.1)	2 (6.5)	
Chemotherapy cycles	1346 (96.3)	126 (94.0)	111 (94.9)	32 (97.0)	38 (92.7)	.52
≤4	52 (3.7)	8 (6.0)	6 (5.1)	1 (3.0)	3 (7.3)	
>4
Sociodemographic and other characteristics						
Race or ethnicity
White	1231 (90.3)	96 (75.6)	87 (77.7)	31 (91.2)	31 (79.5)	<.001^†^
African American	9 (0.7)	3 (2.4)	1 (0.9)	0	3 (7.7)	
Asian	54 (4.0)	11 (8.7)	9 (8.0)	1 (2.9)	1 (2.6)	
Other	69 (5.1)	17 (13.4)	15 (13.4)	2 (5.9)	4 (10.3)	
Education level						
High school or less	109 (7.9)	30 (22.7)	30 (26.8)	8 (23.5)	19 (46.3)	<.001
After high school but not college graduate	305 (22.0)	51 (38.6)	37 (33.0)	5 (14.7)	11 (26.8)	
College or university graduate	635 (45.7)	37 (28.0)	30 (26.8)	13 (38.2)	8 (19.5)	
Postgraduate level	340 (24.5)	14 (10.6)	15 (13.4)	8 (23.5)	3 (7.3)	
Marital status						
Single or never married	392 (28.1)	86 (64.2)	68 (59.1)	0	13 (37.1)	<.001^†^
Married or living as married	914 (65.5)	40 (30.8)	39 (33.9)	32 (100)	15 (42.9)	
Divorced or separated	89 (6.4)	4 (3.1)	8 (7.0)	0	7 (20.0)	
Health insurance coverage						
Yes^‡^	1282 (91.5)	117 (87.3)	100 (85.5)	31 (91.2)	36 (92.3)	.14^†^
No	119 (8.5)	17 (12.7)	17 (14.5)	3 (8.8)	3 (7.7)	
Psychotropic medication use						
Yes	62 (4.4)	8 (6.0)	9 (7.7)	2 (5.9)	10 (24.4)	<.001
No	1339 (95.6)	126 (94.0)	108 (92.3)	32 (94.1)	31 (75.6)	
Engage in vigorous physical activity (≥6 METs)^§^						
Yes	979 (70.1)	78 (58.2)	74 (63.3)	13 (38.2)	16 (39.0)	<.001
No	418 (29.9)	56 (41.8)	43 (36.8)	21 (61.8)	25 (61.0)	
Adverse health outcome^‖^						
Patient-reported hearing loss^¶^						
Grade 0	856 (61.1)	78 (58.2)	72 (61.5)	17 (50.0)	22 (53.7)	.03^†^
Grade 1	347 (24.8)	41 (30.6)	20 (17.1)	7 (20.6)	9 (22.0)	
Grade 2	179 (12.8)	12 (9.0)	23 (19.7)	10 (29.4)	10 (24.4)	
Grade 3	19 (1.4)	3 (2.2)	2 (1.7)	0	0	
Audiometrically assessed hearing loss^#^						
Normal (≤20 dB)	215 (20.4)	39 (38.6)	28 (35.0)	0	3 (11.1)	<.001^†^
Mild (21–40 dB)	267 (25.3)	18 (17.8)	21 (26.3)	0	4 (14.8)	
Moderate (41–55 dB)	157 (14.9)	13 (12.9)	11 (13.8)	2 (7.7)	3 (11.1)	
Moderately severe (56–70 dB)	224 (21.2)	15 (14.9)	11 (13.8)	7 (26.9)	5 (18.5)	
Severe (71–90 dB) or profound (>90 dB)	192 (18.2)	16 (15.8)	9 (11.3)	17 (65.4)	12 (44.4)	
Tinnitus**						
Grade 0	849 (60.6)	70 (52.2)	67 (57.3)	24 (70.6)	21 (51.2)	.005
Grade 1	358 (25.6)	40 (29.9)	31 (26.5)	5 (14.7)	6 (14.6)	
Grade 2	94 (6.7)	16 (11.9)	10 (8.6)	0	6 (14.6)	
Grade 3	100 (7.1)	8 (6.0)	9 (7.7)	5 (14.7)	8 (19.5)	
Peripheral sensory neuropathy^††^						
Grade 0	620 (44.3)	63 (47.0)	41 (35.0)	6 (17.7)	9 (22.0)	<.001
Grade 1	421 (30.1)	34 (25.4)	35 (29.9)	13 (38.2)	9 (22.0)	
Grade 2	196 (14.0)	20 (14.9)	18 (15.4)	6 (17.7)	5 (12.2)	
Grade 3	164 (11.7)	17 (12.7)	23 (19.7)	9 (26.5)	18 (43.9)	
Patient-reported renal disease^‡‡^						
Grade 0	1374 (98.1)	131 (97.8)	113 (96.6)	29 (85.3)	38 (92.7)	<.001^†^
Grade 1 or 2^§§^	27 (1.9)	3 (2.2)	4 (3.4)	5 (14.7)	3 (7.3)	
eGFR-defined renal disease^‖‖^						
Grade 0	386 (48.6)	48 (68.6)	29 (55.8)	5 (29.4)	4 (20.0)	<.001^†^
Grade 1	361 (45.4)	19 (27.1)	18 (34.6)	11 (64.7)	9 (45.0)	
Grade 2	48 (6.0)	3 (4.3)	5 (9.6)	1 (5.9)	7 (35.0)	
CBM_Pt_ score^¶¶^						
None	381 (27.2)	30 (22.4)	28 (23.9)	3 (8.8)	4 (9.8)	<.001^†^
Very low	510 (36.4)	50 (37.3)	38 (32.5)	13 (38.2)	10 (24.4)	
Low	267 (19.1)	27 (20.2)	23 (19.7)	6 (17.7)	5 (12.2)	
Medium	206 (14.7)	26 (19.4)	23 (19.7)	10 (29.4)	18 (43.9)	
High	37 (2.6)	1 (0.8)	5 (4.3)	2 (5.9)	4 (9.8)	
Pain level^##^						
Grade 0	1097 (78.3)	92 (68.7)	72 (61.5)	19 (55.9)	12 (29.3)	<.001
Grade 1	191 (13.6)	21 (15.7)	22 (18.8)	6 (17.7)	4 (9.8)	
Grade 2	104 (7.4)	17 (12.7)	18 (15.4)	8 (23.5)	17 (41.5)	
Grade 3	9 (0.6)	4 (3.0)	5 (4.3)	1 (2.9)	8 (19.5)	
Autonomic neuropathy***						
Grade 0	1050 (75.0)	97 (72.4)	70 (59.8)	23 (67.7)	15 (36.6)	<.001
Grade 1	269 (19.2)	27 (20.2)	26 (22.2)	8 (23.5)	15 (36.6)	
Grade 2	69 (4.9)	8 (6.0)	13 (11.1)	3 (8.8)	3 (7.3)	
Grade 3	13 (0.9)	2 (1.5)	8 (6.8)	0	8 (19.5)	
Self-reported health status^##^						
Excellent	244 (17.5)	22 (16.5)	16 (13.7)	5 (14.7)	1 (2.4)	<.001^†^
Very good	604 (43.3)	42 (31.6)	35 (29.9)	14 (41.2)	6 (14.6)	
Good	491 (35.2)	52 (39.1)	47 (40.2)	10 (29.4)	12 (29.3)	
Fair	55 (3.9)	17 (12.8)	17 (14.5)	4 (11.8)	14 (34.2)	
Poor	2 (0.1)	0	2 (1.7)	1 (2.9)	8 (19.5)	

* Among 1815 TCS included in the cohort, 88 did not provide a response for the question pertaining to current employment status. AHO = adverse health outcome; BEPX3 = 3 cycles of bleomycin, etoposide, and cisplatin; BEPX4 = 4 cycles of bleomycin, etoposide and cisplatin; CBM_Pt_ = cumulative burden of morbidity-platinum; dB = decibel; eGFR = estimated glomerular filtration rate; EOR-CIPN-20 = European Organisation for Research and Treatment of Cancer Chemotherapy-Induced Peripheral Neuropathy 20-item; EP4 = 4 cycles of etoposide and cisplatin; METs = metabolic equivalents; PROs = patient-reported outcomes; PSN = peripheral sensory neuropathy; SCIN = Scale for Chemotherapy-Induced Long-Term Neurotoxicity; TC = testicular cancer; TCS = testicular cancer survivors; VIPX4 = 4 cycles of etoposide, ifosfamide, and cisplatin; VIPX5 = 5 cycles of etoposide, ifosfamide, and cisplatin.

† When 20% or more of cells within a contingency table had expected counts less than five, we used the two-sided Fisher’s exact test to calculate the *P* value. The Fisher exact test was calculated using the fisher.test[stats] function in R (59) with simulate.p.value=T.

‡ Includes those living in Canada who have government-provided health insurance.

§ Physical activity was defined using nine different self-reported activities following previously published methods (55,56).

‖ AHOs are graded according to the definitions in Supplemental Table A1 (available online).

¶ Assessed using the Hearing Handicap Inventory (58) and assessed symptoms at the time of clinical evaluation. For each item, participants were asked to report the age (in years) at first occurrence. If onset of symptoms was before the age of germ cell tumor diagnosis, those responses were not considered when assigning severity grade.

# Among the 1628 participants who provided a response to the question pertaining to current employment status, 1216 underwent audiometric evaluation. Pure-tone air conduction thresholds were obtained bilaterally for each patient at frequencies of 0.25–12 kHz as described previously (13).

** Assessed with the SCIN questionnaire (15).

†† Assessed with the EORTC-CIPN-20 questionnaire (14), the SCIN questionnaire (15), and patient-reported current prescription medication use. Prescription medications were considered only if the respondent stated that the indication was for neuropathy.

‡‡ Assessed using patient-reported information on physician-diagnosed condition and current prescription medication use. Prescription medications were considered only if the respondent stated that the indication was for the AHO of interest.

§§ Only 3 participants reported grade 2 renal disease, and so this group was combined with grade 1.

‖‖ Among the 1727 participants who provided a response to the question pertaining to current employment status, 952 had a serum creatinine measurement. The eGFR was calculated following methods in Levey et al. (18). See Supplemental Table A1 (available online) for details.

¶¶ CBM_Pt_ score using patient-reported outcomes was calculated using patient-reported AHOs previously related to cisplatin exposure (ie, PSN, hearing damage, tinnitus, renal disease) (11).

## Assessed with an item from the SF36 questionnaire (35).

*** Assessed with the EORTC-CIPN-20 questionnaire (14).

**Table 4.** Comparison* of unemployment status between TCS in The Platinum Study and a noncancer population from the BRFSS

	Platinum study*		Normative population (BRFSS)^†^	*P* ^‡^
	Time since chemotherapy completion,	Unemployed	Unemployed	
Age at assessment, y	median [range], y	No. (%)	% (95% CI)	
18–24	1.4 [1.1–3.8]	19 (20.2)	2.2% (1.7 to 2.7)	<.001^‖^
25–29	2.1 [1.0–9.1]	15 (6.8)	2% (1.4 to 2.6)	<.001^‖^
30–34	1.4 [1.0–6.7]	17 (7.0)	1.9% (1.4 to 2.4)	<.001^‖^
35–39	3.9 [1.0–20.1]	9 (3.7)	1.9% (1.4 to 2.4)	<.001^‖^
40–44	2.8 [1.1–4.8]	5 (2.4)	2.2% (1.7 to 2.8)	.04^‖^
45–49	3.5 [1.4–20.1]	7 (4.2)	2.5% (1.8 to 3.3)	.002^‖^
50–54	3.5 [1.1–14.7]	10 (7.3)	3.1% (2.5 to 3.7)	<.001^‖^
55–59^‡^	6.5 [5.1–14.9]	3 (3.7)	3.3% (2.7 to 3.9)	.25

* Restricted to non-Hispanic white men responding “unemployed” when asked about current employment status. BRFSS = Behavioral Risk Factor Surveillance System; CI = confidence interval; TCS = testicular cancer survivors.

† Restricted to non-Hispanic white men with no history of cancer from the Centers for Disease Control and Prevention BRFSS responding “out of work for 1 year or more” when asked about current employment status (https://nccd.cdc.gov/weat/index.html#/crossTabulation/view). BRFSS patients from Guam, the Virgin Islands, and Puerto Rico were excluded.

‡ *P* values are from the Rao-Scott adjusted chi-squaretest (two-sided) using the SAS SURVEYFREQ procedure with the BRFSS sampling weights and a sampling weight of 1 for the Platinum study.

§ No TCS older than 59 years reported being unemployed. Among the BRFSS normative population, 2.6% (95% CI = 2.1% to 3.1%) aged 60–64 years and 0.7% (95% CI = 0.5% to 0.8%) aged 65+ years reported being unemployed.

‖ Statistically significant after controlling for multiple testing false discovery rate. False discovery rate alpha set to 0.05.

After adjusting for statistically significant sociodemographic and clinical characteristics, statistically significantly greater odds of disability leave were associated with PSN (OR = 2.89, *P* = .048, grade 3 vs 0) and reduced eGFR (OR = 12.1, *P* = .01, grade 2 vs 0) (Table 5). CBM_Pt_ score, modeled as a continuous variable, was statistically significantly associated with disability leave (OR = 1.46, 95% CI = 1.03 to 2.08, *P* = .03), with three- and fivefold odds ratios for “medium” and “high” scores, respectively. Pain (not usually associated with cisplatin but strongly correlated here with neuropathy; Pearson *r*^2^ = 0.403, *P* < .001) was also statistically significantly worse among TCS on disability leave vs those employed full-time (OR = 10.59, 95% CI = 4.40 to 25.40, *P* < .001, grade 2 or 3 vs 0). The 95% confidence interval upper limits (UL) were high for eGFR (UL = 70.8, grade 2 vs 0) and pain (UL = 25.4, grade 2 or 3 vs 0), rendering their respective odds ratio estimates (12.07, 10.59) imprecise; however, lower limits indicate strong confidence that population odds ratios are at least 2 and at least 4, respectively.

**Table 5.** Multivariable analyses of AHOs and disability leave* and unemployment^†^ among survivors of cisplatin-treated TC

	On disability leave vs employed full-time		Unemployed vs employed full-time	
	OR		OR	
Variables^‡^	(95% CI)	*P* ***	(95% CI)	*P* ***
CBM_Pt_ score^§^				
None	1.00 (Referent)	—	1.00 (Referent)	—
Very low	1.60 (0.41 to 6.27)	.50	1.04 (0.60 to 1.81)	.89
Low	1.02 (0.21 to 4.94)	.98	1.25 (0.66 to 2.38)	.49
Medium	3.16 (0.79 to 12.6)	.10	1.68 (0.85 to 3.32)	.13
High	5.27 (0.91 to 30.4)	.06	1.84 (0.55 to 6.09)	.32
Patient-reported hearing loss^‖^				
Grade 0	1.00 (Referent)	—	1.00 (Referent)	—
Grade 1	0.68 (0.25 to 1.80)	.43	0.70 (0.39 to 1.25)	.23
Grade 2 or 3^¶^	1.33 (0.52, 3.45)	.55	1.82 (1.04 to 3.17)	.04
Audiometrically assessed hearing loss^#^				
Normal (≤20 dB)	1.00 (Referent)	—	1.00 (Referent)	—
Mild (21–40 dB)	1.34 (0.22 to 8.09)	.67	0.74 (0.38 to 1.44)	.38
Moderate (41–55 dB)	1.75 (0.26 to 11.7)	.51	0.80 (0.34 to 1.85)	.60
Moderately severe (56–70 dB)	1.05 (0.16 to 6.82)	.93	0.65 (0.27 to 1.55)	.33
Severe (71–90 dB) or profound (>90 dB)	3.21 (0.47 to 21.9)	.39	0.77 (0.27 to 2.16)	.62
Tinnitus**				
Grade 0	1.00 (Referent)	—	1.00 (Referent)	—
Grade 1	0.72 (0.24 to 2.09)	.54	0.93 (0.57 to 1.52)	.76
Grade 2	1.31 (0.42 to 4.13)	.64	1.30 (0.61 to 2.79)	.50
Grade 3	2.45 (0.86 to 6.97)	.09	0.95 (0.38 to 2.37)	.91
Peripheral sensory neuropathy^††^				
Grade 0	1.00 (Referent)	—	1.00 (Referent)	—
Grade 1	1.23 (0.43 to 3.54)	.70	1.60 (0.95 to 2.70)	.08
Grade 2	0.83 (0.22 to 3.09)	0.78	1.48 (0.76 to 2.87)	.25
Grade 3	2.89 (1.01 to 8.26)	.048	2.44 (1.28 to 4.62)	.01
Patient-reported renal disease^‡‡^				
Grade 0	1.00 (Referent)	—	1.00 (Referent)	—
Grade 1 or 2^§§^	3.52 (0.71 to 17.3)	.12	2.94 (0.89 to 9.69)	.08
eGFR-defined renal disease^‖‖^				
Grade 0	1.00 (Referent)	—	1.00 (Referent)	—
Grade 1	1.18 (0.26 to 5.30)	.75	0.81 (0.40 to 1.63)	.55
Grade 2	12.1 (2.06 to 70.8)	.01	1.76 (0.50 to 6.23)	.38
Pain^¶¶^				
Grade 0	1.00 (Referent)	—	1.00 (Referent)	—
Grade 1	1.28 (0.33 to 5.03)	.72	1.78 (1.00 to 3.15)	.05
Grade 2 or 3^##^	10.6 (4.40 to 25.4)	<.001	3.75 (2.06 to 6.81)	<.001

* Each row of analysis is derived from a multivariable logistic regression model of disability leave vs employed full-time (reference group) in which we report the effect of the primary independent variable of interest listed in the table adjusted for covariates identified on backward model selection: age at evaluation, time since chemotherapy completion, educational status, marital status, and use of psychotropic medications. Because no statistically significant interactions were found between time since chemotherapy and any of the AHOs, no interaction terms were included in the models. Enrollment site was not included as an adjustment factor, because 2 sites had no individuals who reported being on disability; however, sensitivity analysis confirmed that exclusion of these sites from the analysis did not alter the conclusion for any of the models. AHO = adverse health outcome; CBM_Pt_ = cumulative burden of morbidity-platinum; CI = confidence interval; eGFR = estimated glomerular filtration rate; EORTC-CIPN-20 = European Organisation for Research and Treatment of Cancer Chemotherapy-Induced Peripheral Neuropathy 20-item; OR = odds ratio; PSN = peripheral sensory neuropathy; Ref = reference group; SCIN = Scale for Chemotherapy-Induced Long-Term Neurotoxicity; TC = testicular cancer.

† Each row of analysis is derived from a multivariable logistic regression model of unemployed vs employed full-time (reference group) in which we report the effect of the primary independent variable of interest listed in the table adjusted for covariates identified on backward model selection: age at evaluation, time since chemotherapy completion, educational status, marital status, and use of psychotropic medications. Enrollment site was also included as a prespecified covariate. Because no statistically significant interactions were found between time since chemotherapy and any of the AHOs, no interaction terms were included in the models.

‡ AHOs are graded according to the definitions in Supplemental Table A1 (available online).

§ CBM_Pt_ score was calculated using patient-reported AHOs previously related to cisplatin exposure (ie, PSN, hearing loss, tinnitus, renal disease) using a modification of Kerns et al. (11).

‖ Assessed using the Hearing Handicap Inventory (58) administered at the time of clinical evaluation. For each item, participants were asked to report the age (in years) at first occurrence. If onset of symptoms was before the age of germ cell tumor diagnosis, those responses were not considered when assigning severity grade.

¶ Only 24 participants reported grade 3 hearing loss, and so this group was combined with grade 2.

# Pure-tone air conduction thresholds were obtained bilaterally for each patient at frequencies of 0.25–12 kHz as described previously (13).

** Assessed with the SCIN questionnaire (15) based on symptoms experienced over the past 4 weeks.

†† Assessed with the EORTC-CIPN-20 questionnaire (14), the SCIN questionnaire (15), and patient-reported current prescription medication use. Prescription medications were only considered if the respondent stated that the indication was for neuropathy.

‡‡ Assessed using patient-reported information on physician-diagnosed condition and current prescription medication use. Prescription medications were considered only if the respondent stated that the indication was for the AHO of interest.

§§ Only 3 participants reported grade 2 renal disease, and so this group was combined with grade 1.

‖‖ The eGFR was calculated following methods in Levey et al. (18). See Supplemental Table A1 (available online) for details.

¶¶ Assessed with an item from the SF36 questionnaire (35).

## Only 8 participants reported grade 3 pain and disability leave, and 9 participants reported grade 3 pain and unemployment; thus, grade 3 was combined with grade 2 in both models.

*** *P* values are from a Wald test and are two-sided.

Table 5 also shows associations between AHOs and unemployment, adjusting for sociodemographic and clinical characteristics statistically significantly associated with unemployment. Although CBM_Pt_ score was not statistically significantly associated with unemployment, individual AHOs including patient-reported hearing loss (OR = 1.82, 95% CI = 1.04 to 3.17, grade 2 or 3 vs 0, *P* = .04), PSN (OR = 2.44, 95% CI = 1.28 to 4.62, grade 3 vs 0, *P* = .006), and pain (OR = 3.75, 95% CI = 2.06 to 6.81, grade 2 or 3 vs 0, *P* < .001) were associated with increased odds of unemployment.

Increasing severity of most cisplatin-related AHOs was associated with statistically significantly worse SRH after adjusting for SRH-related sociodemographic factors in the general population (see “Methods”) (36–40) (Table 6). All PSN grades were statistically significantly associated with worse SRH (OR = 1.26, 95% CI = 1.01 to 1.58; OR = 2.77, 95% CI = 2.06 to 3.71; and OR = 2.50, 95% CI = 1.84 to 3.39 for grades 1, 2, and 3 vs 0, respectively, *P* < .05 each) as was pain (*P* ≤ .001 for each grade vs grade 0). The OR for pain (grade 3 vs 0) was imprecisely estimated; however, the lower limits suggest strong confidence that the population odds of worse SRH are at least 5 times for grade 3 vs 0. Patient-reported hearing loss (OR = 1.42, 95% CI = 1.14 to 1.77; and OR = 2.29, 95% CI = 1.74 to 3.01, grades 1 and 2 or 3 vs 0, respectively; *P* < .01 each), audiometrically assessed hearing loss (OR = 1.54, 95% CI = 1.03 to 2.31 for severe to profound vs normal, *P* = .04), and patient-reported renal disease (OR = 3.14, 95% CI = 1.74 to 5.67, grade 1 or 2 vs 0, *P* = .001) were also statistically significantly associated with worse SRH.

**Table 6.** Multivariable analyses* of individual AHOs^†^ and SRH status

	OR	*P*
Adverse health outcomes	(95% CI)	
Patient-reported hearing loss^‡^		
Grade 0	1.00 (Referent)	—
Grade 1	1.42 (1.14 to 1.77)	.002
Grade 2 or 3^§^	2.29 (1.74 to 3.01)	<.001
Audiometrically assessed hearing loss^‖^		
Normal (≤20 dB)	1.00 (Referent)	—
Mild (21–40 dB)	1.41 (1.02 to 1.94)	.04
Moderate (41–55 dB)	1.26 (0.87 to 1.82)	.22
Moderately severe (56–70 dB)	1.10 (0.77 to 1.58)	.60
Severe (71–90 dB) or profound (>90 dB)	1.54 (1.03 to 2.31)	.04
Tinnitus^¶^		
Grade 0	1.00 (Referent)	—
Grade 1	1.29 (1.04 to 1.60)	.02
Grade 2	2.68 (1.86 to 3.85)	<.001
Grade 3	1.36 (0.95 to 1.95)	.09
Peripheral sensory neuropathy^#^		
Grade 0	1.00 (Referent)	—
Grade 1	1.26 (1.01 to 1.58)	.04
Grade 2	2.77 (2.06 to 3.71)	<.001
Grade 3	2.50 (1.84 to 3.39)	<.001
Patient-reported renal disease**		
Grade 0	1.00 (Referent)	—
Grade 1 or 2^††^	3.14 (1.74 to 5.67)	.001
eGFR-defined renal disease^‡‡^		
Grade 0	1.00 (Referent)	—
Grade 1	1.01 (0.77 to 1.31)	.97
Grade 2	1.48 (0.88 to 2.50)	.14
Pain^§§^		
Grade 0	1.00 (Referent)	—
Grade 1	2.19 (1.67 to 2.87)	<.001
Grade 2	4.78 (3.38 to 6.77)	<.001
Grade 3	12.7 (5.75 to 27.9)	<.001

* Each row of analysis is derived from a multivariable ordinal regression model in which we report the effect for the primary independent variable of interest after adjustment for enrollment center as well as covariates related to SRH in the general population: age, race, educational level, employment status, smoking status, and physical activity (36–40). Please refer to Methods. AHO = adverse health outcome; CI = confidence interval; eGFR = estimated glomerular filtration rate; EORTC-CIPN-20 = European Organisation for Research and Treatment of Cancer Chemotherapy-Induced Peripheral Neuropathy 20-item; OR = odds ratio; Ref = reference group; SCIN = Scale for Chemotherapy-Induced Long-Term Neurotoxicity; SRH = self-reported health.

† All AHOs are based on patient-reported outcomes, unless otherwise stated, and are graded according to the definitions in Supplemental Table A1 (available online).

‡ Assessed using the Hearing Handicap Inventory (58) and assessed symptoms at the time of clinical evaluation. For each item, participants were asked to report the age (in years) at first occurrence. If onset of symptoms was before the age of germ cell tumor diagnosis, those responses were not considered when assigning severity grade.

§ Only 20 participants reported grade 3 hearing loss and so were combined with those reporting grade 2 hearing loss.

‖ Pure-tone air conduction thresholds were obtained bilaterally for each patient at frequencies of 0.25–12 kHz as described previously (13).

¶ Assessed with the SCIN questionnaire (15).

# Assessed with the EORTC-CIPN-20 questionnaire (14), the SCIN questionnaire (15), and patient-reported current prescription medication use. Prescription medications were only considered if the respondent stated that the indication was for neuropathy.

** Assessed using patient-reported information on physician-diagnosed condition and current prescription medication use. Prescription medications were considered only if the respondent stated that the indication was for the AHO of interest.

†† Only 3 participants reported grade 2 renal disease (defined as renal disease requiring prescription medication) and so were combined with those reporting grade 1 renal disease (defined as renal disease without prescription medication).

V The eGFR was calculated following methods in Levey et al. (18). See Supplemental Table A1 (available online) for details.

§§ Assessed with an item from the SF36 questionnaire (35).

## Discussion

In the largest study of TCS to date, we describe for the first time, to our knowledge, employment outcomes in relationship to cisplatin-related AHOs. Importantly, our well-characterized population is largely US based and shows that at a median of approximately 4 years after treatment, about 1 in 10 TCS is either on disability leave or unemployed. Statistically significantly higher odds of disability leave were associated with increasing grades of PSN, objectively assessed renal disease, increasing CBM_Pt_ score, and pain. Overall, a statistically significantly greater proportion of TCS was unemployed compared with a normative US population, with associated AHOs, including neuropathy, patient-reported hearing loss, and pain. SRH was markedly worse among unemployed TCS and those on disability leave vs TCS employed full-time. These and other new findings are discussed below.

Data on relationships between cancer and its treatment and employment status derive largely from studies of childhood cancer survivors or survivors of cancers with an older average age at onset than TC. Statistically significantly greater percentages of participants in the Childhood Cancer Survivor Study had ever received supplemental security income (10%) or social security disability insurance (14%) compared with noncancer survivors (2.6% and 5.4%, respectively); the presence of at least one severe or life-threatening health condition increased risk of supplemental income and disability insurance by 3.8- and 2.7-fold, respectively (6). More childhood cancer survivors also reported greater health-related unemployment (10.4%) vs siblings (1.8%) (42). Data from the U.S. National Health Interview Survey showed that adult-onset cancer survivors were statistically significantly more likely to be unable to work because of a health condition than individuals without cancer histories (43). Studies of adult-onset European cancer survivors also demonstrated an association between increasing age (10), lower educational status (10), and increased disability leave, which are supported by our results. We also show a relationship with non-white ethnicity.

Prior investigations of disability and sick leave among TCS to date have largely been conducted in Europe without evaluations of treatment-associated AHOs (5,7–10). In contrast, we directly assessed relationships between cisplatin-related AHOs and disability leave. A statistically significantly greater percentage of TCS on disability leave experienced grade 2 or 3 cisplatin-related AHOs (24%) vs those employed full-time (15%, *P* < .001). In particular, statistically significant roles for higher grades of neuropathy and objectively defined renal disease were associated with increased disability leave, and these TCS were more likely to use prescription antipsychotropic medications (24%) than those employed full-time (4%, *P* < .001). No agents are approved to prevent or treat chemotherapy-induced peripheral neuropathy, and there is only a moderate recommendation for duloxetine to treat related pain (44). In the general population, renal dysfunction is associated with statistically significantly increased eightfold risks of labor force nonparticipation, even after exclusion of patients with markedly elevated creatinine levels (>4 mg/dL [men], >3.7 mg/dL [women]) (45).

Although US TCS data are sparse, insights can be gained from studies of adolescent and young adult (AYA) cancer survivors aged 15–39 years at diagnosis (46–48). Analyses of US Medical Expenditure Panel Survey data showed that AYA survivors were more likely than adults without cancer histories to be unable to work because of illness or disability (47). Further, they experienced excess annual productivity losses (including employment disability, fewer hours worked, and more missed work days) of $2250 per person (47). Results from National Cancer Institute's AYA-Health Outcomes and Patient Experience study showed that among survivors who were employed or in school full-time at diagnosis, factors associated with employment status at follow-up (range = 15–35 months postdiagnosis) included treatment intensity and comorbidity score, although the latter excluded treatment-related toxicities (48). Another analysis of US Medical Expenditure Panel Survey data showed that even among employed AYA cancer survivors, cancer and its treatment interfered with on-the-job tasks, suggesting the percentage of survivors on disability leave may underestimate the true deleterious impact on employment (46).

Nonetheless, it remains difficult to isolate the contributions of a TC diagnosis and cisplatin-related AHOs to unemployment status. Reasons for unemployment are mixed and may include young age with ongoing continuing education, TC-related educational delays, and work interruption. However, using BRFSS normative data, we showed across a number of age groups that a statistically significantly greater proportion of TCS are unemployed vs men of similar age, race, and ethnicity. This finding is consistent with studies in other cancer survivor populations. For example, among adult-onset cancer survivors in the United States and Europe, a meta-analysis showed that cancer survivors were more likely to be unemployed than matched healthy population controls (33.8% vs 15.2%, pooled relative risk = 1.37, 95% CI = 1.21 to 1.55) (49).

Worse SRH was seen in TCS with cisplatin-related AHOs, including hearing loss, tinnitus, PSN, and patient-reported renal disease. Interestingly, objectively measured renal disease was not associated with worse SRH, suggesting that subclinical renal dysfunction may not affect perceived health status. Pain was also strongly associated with worse SRH and was highly correlated with neuropathy (*P* < .001). In the general US population, poorer SRH is associated with shorter life expectancy (38) and increased mortality independent of clinical and physician assessments (50). Thus, formal evaluations of SRH among TCS during follow-up visits could provide useful information along with standard clinical assessments.

Our findings support BRFSS results in which respondents from the general population with at least 3 chronic health conditions had the highest risk of reporting fair or poor health compared with those with no chronic conditions (adjusted OR = 8.7, 95% CI = 8.0 to 9.4) (37). Data on health conditions related to cisplatin-based chemotherapy and relationships with SRH, however, are lacking. Among AYA survivors, including TC, those with at least 2 comorbidities based on the Young Adult HOPE-Index were statistically significantly more likely to report fair or poor SRH (OR = 3.16, 95% CI = 1.58 to 6.33) than those with no comorbidities (51). However, comorbidity was defined after excluding all treatment-related toxicities. Our study thus addresses an important gap by identifying, specifically, cisplatin-related AHOs associated with SRH among a relatively young group of cancer survivors. Other AHOs not included in our CBM_Pt_ score such as cardiovascular disease (52,53) and those related to metabolic syndromes (54) may also relate in part to cisplatin exposure; however, it is difficult to isolate cisplatin’s contribution from genetic and lifestyle factors.

Major strengths of our study include the large cohort size, detailed medical record abstraction, physical examinations, laboratory-based measures, and use of PROs. Collection of sociodemographic data allowed for adjustment of factors related to disability leave and SRH status in the general population (36–40). Any cross-sectional design has inherent limitations and does not permit assignment of causation of evaluated risk factors to outcomes, noting that employment status at TC diagnosis or chemotherapy initiation was not queried. In addition, enrollment of study participants from major academic cancer centers may limit generalizability of findings. Employment status was assessed during study enrollment concurrently with audiometry or other procedures and thus provides an accurate snapshot of the patient at that point in time. However, to decrease patient burden and in view of the short median follow-up, employment history, including job type, was not queried. Thus, it is unclear whether TCS were on long-term or short-term disability or whether unemployment was health related, although given their young median age, any workforce interruption is concerning.

In conclusion, at a median of approximately 4 years after completing treatment, most TCS are employed and report good or better health. However, a small group experiences multiple cisplatin-related AHOs, often severe, which are associated with disability leave, unemployment, and worse SRH. These findings have important implications with regard to losses in productivity and socioeconomic costs in this relatively young population. An awareness of AHOs associated with disability leave can help focus efforts in developing interventions and strategies to ameliorate or prevent these outcomes. Our finding that cisplatin-related AHOs are perceptible to patients and are associated with SRH highlights the importance of incorporating PROs in survivorship care strategies.

### Funding

This work was supported by the National Cancer Institute (1R01 CA157823 to LBT and K07 CA187546 to SLK). RH is supported by the National Institute for Health Research Institute of Cancer Research and Royal Marsden Biomedical Research Centre and Josh Carrick Foundation.

### Notes

**Role of the funder:** The funder was not involved in the design of the study; the collection, analysis, and interpretation of the data; the writing of the manuscript; and the decision to submit the manuscript for publication.

**Disclosures:** Chunkit Fung served on advisory panels for Novartis and Exelixis but the work related to these advisory panels presents no conflict of interest with research related to this manuscript.

## Supplementary Material

pkaa022_Supplementary_Data
