# Supplementary material for: Relationship of Cisplatin-Related Adverse Health Outcomes With Disability and Unemployment Among Testicular Cancer Survivors
Source: JNCI Cancer Spectr. 2020 Mar 20;4(4):pkaa022. doi: 10.1093/jncics/pkaa022 (PMC7368467; doi:10.1093/jncics/pkaa022)
Supplement: pkaa022_Supplementary_Data [file pkaa022_supplementary_data.pdf]

SUPPLEMENTARY MATERIALS

Study Population

All participants were administered first-line cisplatin-based chemotherapy for either initial germ cell tumor or recurrence after active surveillance. Participants could not have received radiotherapy, undergone bone marrow transplantation, or received antecedent chemotherapy for another primary cancer. All participants were undergoing routine follow-up at the participating site, and at the time of clinical assessment, were disease-free. Enrolling sites were located in the U.S. (University of Rochester, University of Pennsylvania, Indiana University, Dana Farber Cancer Institute, Memorial Sloan Kettering Cancer Center, M.D. Anderson), Canada (Princess Margaret Hospital and British Columbia Cancer Agency), and the Royal Marsden Hospital in England.

Medical Record Abstraction

Study personnel from all enrolling sites were trained centrally in the abstraction of medical record data, using a standard protocol and forms adapted from prior analytic investigations [60-63]. Detailed data describing cancer diagnosis and therapy, including names and doses of all cytotoxic drugs were collected directly from medical records.

Sociodemographic Characteristics, Health Behaviors and Patient-Reported Outcomes

Sociodemographic characteristics, health behaviors, and patient-reported outcomes (PROs), were assessed through self-reporting using validated questionnaires [14,15,35,56-58]. We applied strict definitions to assessment times to minimize recall bias.

For sociodemographic characteristics, we assessed marital and employment status and educational level at the time of enrollment, as well as self-reported race. Normative data on unemployment were ascertained from the 2016 Centers for Disease Control and Prevention

Behavioral Risk Factor Surveillance System (BRFSS) [22], accessed October 2018) and were used to compare unemployment rates by age groups between testicular cancer survivors (TCS) and the general U.S. population, matching on race/ethnicity. BRFSS is a cross-sectional, random-digit–dialed telephone survey of >400,000 non-institutionalized U.S. adults aged ≥18 years in the 50 states, the District of Columbia (DC), Puerto Rico, the U.S. Virgin Islands, and Guam that uses trained interviewers and standard core and optional questionnaire modules to collect uniform data (details at http://www.cdc.gov/brfss/index.htm). Surveys are conducted with U.S. households each month using disproportionate, stratified, random sampling in all states and Washington D.C. and simple random sampling in Guam, Puerto Rico, and the U.S. Virgin Islands. Employment status in BRFSS is assessed as follows: "Are you currently?: Employed for wages; Self-employed; Out of work for 1 year or more; Out of work for less than 1 year; A homemaker; A student; Retired; Unable to work; Refused; Not asked or Missing". Responses of "Out of work for 1 year or more" were used for comparison to unemployed TCS. Analyses excluded BRFSS participants from Guam, Puerto Rico, and the U.S. Virgin Islands.

To assess health behaviors, we used validated questionnaires to query current or former smoking status, alcohol consumption, and physical activity over the past year. The validated physical activity questionnaire [57,58] asked participants to report their average time per week (over the past year) spent at each of nine recreational activities: walking or hiking (including walking to work); jogging (slower than 10-minute miles); running (10- minute miles or faster); bicycling (including stationary bike); aerobic exercise or dance/exercise machines; lower intensity exercise/yoga/stretching/toning; tennis, squash or racquetball; lap swimming; and weight lifting/strength training. Each physical activity was assigned a metabolic equivalent (MET) value. MET values are a commonly used metric for describing the relative energy expenditure of a specific type of physical activity (1 MET=1 kcal/kg/hour, or the energy cost of sitting quietly). The MET values for each activity were then used to calculate MET-hours/week for each participant, and these were

grouped into categories of vigorous or non-vigorous physical activity applying standard definitions [56].

Patient-reported adverse health outcomes (AHOs) were assessed at the time of clinical evaluation using validated questionnaires including the European Organization for Research and Treatment of Cancer Chemotherapy-Induced Peripheral Neuropathy (EORTC CIPN-20) [14], the Scale for Chemotherapy-Induced Neurotoxicity (SCIN) [15] and the Hearing Handicap Inventory [58]. For those AHOs for which Common Terminology Criteria for Adverse Events (CTCAE v4.03) [16] grading took into account current prescription medication use (i.e. peripheral sensory neuropathy, pain, kidney disease, hypercholesterolemia, hypertriglyceridemia, hypertension, diabetes, peripheral artery disease, thromboembolic event, thyroid disease, anxiety/depression, erectile dysfunction, and hypogonadism), we only took into account current prescription medication use (with usage for more than one month), using data provided by the patient at the time of clinical evaluation. Pearson's correlation coefficient (r^2) was used to assess the correlation between patient-reported peripheral sensory neuropathy and pain.

<u>Objective Assessment of AHOs</u>

Body mass index (BMI) was derived from height and weight assessed at the time of clinical evaluation using the following equation: weight (kg)/height $(m)^2$. Audiometric data assessed at 0.25, 0.5, 1, 1.5, 2, 3, 4, 6, 8, 10 and 12 kHz were categorized according to the severity of hearing loss as defined previously [13] following American Speech-Language-Hearing Association criteria [17]. Kidney disease was based on the estimated glomerular filtration rate (eGFR) that is used in clinical practice guidelines [64]; this was calculated with serum creatinine measured at the time of clinical evaluation, using the CKD-EPI equation described in Levey et al [18]. This equation is as follows:

$$eGFR = 141 \times \min(Scr/\kappa,1)^{\alpha} \times \max(Scr/\kappa,1)^{-1.209} \times 0.993^{Age} \times 1.018[\text{if female}] \times 1.159[\text{if black}]$$

Where:

Scr = serum creatinine (mg/dL)

κ = 0.7 if female; 0.9 if male

α = -0.329 if female; -0.411 if male

min = the minimum of Scr/κ or 1

max = the maximum of Scr/κ or 1

Statistical Analysis

The following were considered as potential explanatory (independent) variables in multivariable logistic regression models of employment status (dependent variable) reported in Table 5: time since end of chemotherapy, attained age at clinical assessment, number of chemotherapy cycles, race/ethnicity, educational status, marital status, health insurance status, current use of anti-psychotropic medication use, and engagement in vigorous physical activity. All were considered in the full model and backward model selection chose variables with $p<0.05$ for retention as adjustment factors in the final model.

Supplementary Table 1. Grading of Adverse Health Outcomes[*].

Adverse Health Outcome	Platinum Study item(s) used to assign severity grade	Grade 1	Grade 2	Grade 3	Grade 4
Peripheral sensory neuropathy	Tingling fingers/hands or toes/feet[†]<br>Numbness in fingers/hands or toes/feet[†]<br>Shooting or burning pain in fingers/hands or toes/feet[†]<br>Difficulty distinguishing between hot and cold water[†]<br>Problems standing/walking because of difficulty feeling ground under feet[†]<br>Pain and tingling in fingers/hands or toes/feet[§]<br>Prescription medication use[#]	A little	Quite a bit	Very much	NA[‡]
Autonomic neuropathy	Dizzy when standing up from a sitting or lying position[†]	A little	Quite a bit	Very much	NA
Patient reported hearing loss	Difficulty hearing[†]<br>Reduced hearing[§]<br>Hearing loss requiring a hearing aid[‖]<br>Complete deafness[‖]	A little (difficulty hearing or reduced hearing)	Quite a bit or very much (difficulty hearing or reduced hearing)	Hearing loss requiring a hearing aid in one or both ears OR complete deafness in one ear	Complete deafness in both ears
Tinnitus	Ringing in ears[§]	A little	Quite a bit	Very much	NA
Raynaud phenomenon	White cold fingers/hands or toes/feet when it is cold[§]	A little	Quite a bit	Very much	NA
Pain	How much did pain interfere in normal work (including work outside the home and inside the house, yard)[¶]<br>Prescription medication use[#]	A little bit	Moderately OR quite a bit OR medication use	Extremely	NA
Patient reported kidney disease	Told by doctor of condition<br>Prescription medication use[#]	Have condition	Have condition AND medication use	NA	NA
eGFR-defined kidney disease	eGFR calculation using serum creatinine measurement[**]	60-89 mL/min/1.73 m^2	30-59 mL/min/1.73 m^2	15-29 mL/min/1.73 m^2	<15 mL/min/1.73 m^2
Hyper-cholesterolemia	Told by doctor of condition<br>Prescription medication use for high total cholesterol or low HDL cholesterol[#]	NA	Have condition AND medication use	NA	NA
Hyper-triglyceridemia	Prescription medication use[#]	NA	Medication use for condition	NA	NA
Hypertension	Told by doctor of condition<br>Prescription medication use[#]	NA	Have condition AND medication use	NA	NA

**Supplementary Table 1 continued.** Grading of Adverse Health Outcomes*					
**Adverse Health Outcome**	**Platinum Study item(s) used to assign severity grade**	**Grade 1**	**Grade 2**	**Grade 3**	**Grade 4**
Diabetes	Told by doctor of condition<br>Prescription medication use#	NA	Have condition AND taking tablets or pills	Have condition AND taking insulin	NA
Coronary artery disease	Told by doctor of condition - angina<br>Told by doctor of condition - coronary artery disease<br>Heart attack or myocardial infarction<br>Had relevant procedure	Have angina or coronary artery disease	Have angina or coronary artery disease AND either medication use, angioplasty, or stent placement	Had heart attack or myocardial infarction OR have had coronary bypass surgery	NA
Transient ischemic attack	Told by doctor of condition	Have condition	NA	NA	NA
Stroke	Told by doctor of condition<br>Surgical procedure	NA	Have condition	NA	Have condition AND had carotid artery surgery
Peripheral artery disease	Pain in calf when walking (intermittent claudication)<br>Had relevant procedure<br>Prescription medication use#	Have condition	Have condition AND medication use	Have condition AND had peripheral artery surgery	NA
Thromboembolic event	Blood clot in leg (deep vein thrombosis)<br>Blood clot in lung (pulmonary embolism)<br>Prescription medication use#	NA	Have deep vein thrombosis	Have pulmonary embolism OR medication use	NA
Obesity	Body mass index††	NA	BMI: 25-29 kg/m^2	BMI:30-39 kg/m^2	BMI ≥ 40 kg/m^2
Thyroid disease	Overactive thyroid<br>Underactive thyroid<br>Prescription medication use#	Have condition	Have condition AND medication use	NA	NA
Anxiety/depression	Prescription medication use#	NA	Medication use for condition	NA	NA
Erectile dysfunction	Difficulty getting or maintaining an erection†<br>Prescription medication use**	A little	Quite a bit or very much OR medication use	NA	NA
Hypogonadism	Prescription medication use#	NA	Medication use for condition	NA	NA

* Table is modified from Kerns SL et al. [11] with the addition of estimated glomerular filtration rate (eGFR)-defined kidney disease [18]. For conditions based on more than one question, the severity grade was assigned based on the response reporting the greatest or most severe symptom.
† Assessed with the European Organisation for Research and Treatment of Cancer Chemotherapy-Induced Peripheral Neuropathy 20-item (EORTC/CIPN-20) questionnaire [14].
‡ NA, not applicable. Data needed to assign grade were not captured.
§ Assessed with the Scale for Chemotherapy-Induced Long-Term Neurotoxicity (SCIN) questionnaire [15].
|| Item is from the Hearing Handicap Inventory by Ventry and Weinstein and assessed symptoms at the time of clinical evaluation [59]. For each item, participants were asked to report the age (in years) at first occurrence. If onset of symptoms was prior to age of TC diagnosis, those responses were not considered when assigning severity grade.
¶ Item is from the SF36 questionnaire [35].
# Prescription medications taken for at least the past 4 weeks were only used to assign grade if the participant reported that the indication was for the given adverse health outcome and the medication was started during or after chemotherapy.
** eGFR was calculated using the following formula for males: $eGFR = 141 \times \min(S_{Cr}/0.9, 1)^{-0.411} \times \max(S_{Cr}/0.9, 1)^{-1.209} \times 0.993^{Age} \times 1.159$ [if Black], where S_{Cr} is standardized serum creatinine (in mg/dL), min indicates the minimum of $S_{Cr}/0.9$ or 1, max indicates the maximum of $S_{Cr}/0.9$ or 1, and age is in units of years [18].
†† Body mass index is based on physical examination performed at time of clinical assessment.

Supplementary Table 2. Definition of the Cumulative Burden of Morbidity-Platinum (CBM_{Pt}) Score Based on Number and Severity of Individual Platinum-Related Adverse Health Outcomes*

	**None**	**Very Low**	**Low**	**Medium**	**High**
Grade 1	0	≥ 1	Any number	Any number	Any number
Grade 2	0	0	≥ 1	Any number	Any number
Grade 3	0	0	0	≥ 1	≥ 2
Grade 4	0	0	0	0	0

* Methods adapted from Geenen et al[21] as defined in Kerns et al [11]. Modifications include division of the low category into very low and low, and division of the high category into high and very high to reflect the granularity of data collected in the present study. Audiometrically assessed hearing loss was not included, as it was categorized following American Speech-Language-Hearing Association criteria guidelines [17] and thus, not assigned a Common Terminology Criteria for Adverse Events (CTCAEv4.03) grade [16].

Supplementary Table 3. Prevalence of Individual Adverse Health Outcomes (AHOs) and CBM_{Pt} Score by Treatment Regimen[*]					
	**BEPX3 N = 644**	**EPX4 N = 540**	**BEPX4 N = 308**	**VIPX4/ VIPX5 N = 46**	**Cisplatin-based chemotherapy ≥ 5 cycles† N = 78**
AHOs, N (%)					
Number of AHOs of any grade‡					
0	51 (7.9)	24 (4.4)	19 (6.2)	3 (6.5)	4 (5.1)
1	109 (16.9)	81 (15.0)	38 (12.3)	7 (15.2)	8 (10.3)
2	87 (13.5)	110 (20.4)	34 (11.0)	5 (10.9)	10 (12.8)
3	110 (17.1)	89 (16.5)	54 (17.5)	8 (17.4)	12 (15.4)
4	108 (16.8)	79 (14.6)	49 (15.9)	6 (13.0)	10 (12.8)
5+	179 (27.8)	157 (29.1)	114 (37.0)	17 (37.0)	34 (43.6)
CBM_{Pt} score§					
None	193 (30.0)	152 (28.2)	68 (22.1)	14 (30.4)	14 (18.0)
Very low	247 (38.4)	179 (33.2)	103 (33.4)	11 (23.9)	22 (28.2)
Low	101 (15.7)	98 (18.2)	74 (24.0)	11 (23.9)	16 (20.5)
Medium	91 (14.1)	96 (17.8)	56 (18.2)	7 (15.2)	19 (24.4)
High	12 (1.9)	15 (2.8)	7 (2.3)	3 (6.5)	7 (9.0)
Peripheral sensory neuropathy‖					
Grade 0	306 (47.5)	230 (42.6)	119 (38.6)	21 (45.7)	32 (41.0)
Grade 1	194 (30.1)	149 (27.6)	92 (29.9)	9 (19.6)	18 (23.1)
Grade 2	75 (11.7)	81 (15.0)	54 (17.5)	7 (15.2)	10 (12.8)
Grade 3	69 (10.7)	80 (14.8)	43 (14.0)	9 (19.6)	18 (23.1)
Tinnitus¶					
Grade 0	409 (63.5)	327 (60.6)	177 (57.5)	23 (50.0)	35 (44.9)
Grade 1	155 (24.1)	146 (27.0)	79 (25.7)	15 (32.6)	19 (24.4)
Grade 2	39 (6.1)	28 (5.2)	29 (9.4)	4 (8.7)	12 (15.4)
Grade 3	41 (6.4)	39 (7.2)	23 (7.5)	4 (8.7)	12 (15.4)
Patient-reported hearing loss[#]					
Grade 0	413 (64.1)	331 (61.3)	188 (61.0)	25 (54.4)	39 (50.0)
Grade 1	148 (23.0)	139 (25.7)	73 (23.7)	9 (19.6)	18 (23.1)
Grade 2	77 (12.0)	63 (11.7)	42 (13.6)	12 (26.1)	18 (23.1)
Grade 3	6 (0.9)	7 (1.3)	5 (1.6)	0	3 (3.9)
Audiometrically-defined hearing loss[**]					
Normal ≤20 dB	122 (24.3)	74 (18.1)	50 (23.6)	11 (30.6)	6 (14.6)
Mild 21-40 dB	130 (25.8)	107 (26.1)	37 (17.5)	7 (19.4)	4 (9.8)
Moderate 41-55 dB	76 (15.1)	49 (12.0)	38 (17.9)	4 (11.11)	5 (12.2)
Moderately severe 56-70 dB	97 (19.3)	78 (19.0)	53 (25.0)	4 (11.11)	16 (39.0)
Severe 71-90 dB	68 (13.5)	92 (22.4)	33 (15.6)	9 (25.0)	7 (17.1)
Profound >90 dB	10 (2.0)	10 (2.4)	1 (0.5)	1 (2.8)	3 (7.3)
Estimated glomerular filtration rate, $mL/min/1.73m^2$ ††					
Normal or high ≥90	218 (58.3)	129 (43.0)	87 (47.5)	12 (41.4)	6 (35.3)
Mild reduction 60-89	142 (38.0)	147 (49.0)	82 (44.8)	13 (44.8)	9 (52.9)
Mild to moderate reduction 45-59	11 (2.9)	22 (7.3)	8 (4.4)	3 (10.3)	0
Moderate to severe reduction 30-44	3 (0.8)	2 (0.7)	6 (3.3)	1 (3.5)	2 (11.8)

Abbreviations: BEPX3, 3 cycles of bleomycin, etoposide, and cisplatin; BEPX4, 4 cycles of bleomycin, etoposide, and cisplatin; CBM_{Pt}, cumulative burden of morbidity-platinum; EPX4, 4 cycles of etooside and cisplatin; VIPX4, 4 cycles of etoposide, ifosfamide, and cisplatin; VIPX5, 5 cycles of etoposide, ifosfamide, and cisplatin.

*[a] Results (percentages) are also depicted graphically in Figure 1.

†[b] Includes 5 cycles (n=29), 6 cycles (n=38), 7 cycles (n=5), and 8 or more cycles (n=6) of cisplatin-based chemotherapy. All chemotherapy was completed ≥1 year before study entry (see Methods).

‡[c] Patient-reported outcomes are used to define all variables except obesity, which is based on body mass index measured at time of clinical assessment.

§[d] CBM_{Pt} score was calculated using patient-reported adverse health outcomes previously related to cisplatin exposure (i.e. peripheral sensory neuropathy, hearing loss, tinnitus, renal disease) using a modification of Kerns et al.[11]

|| [e] Assessed with the European Organisation for Research and Treatment of Cancer Chemotherapy-Induced Peripheral Neuropathy 20-item (EORTC/CIPN-20) questionnaire,[14] the Scale for Chemotherapy-Induced Long-Term Neurotoxicity (SCIN) questionnaire,[15] and patient-reported current prescription medication use. Prescription medications were only considered if the respondent stated that the indication was for neuropathy.
¶ [f] Assessed with the SCIN questionnaire[15] based on symptoms experienced over the past 4 weeks. If participants responded that the symptom started before chemotherapy, those responses were not considered when assigning severity grade.
# [g] Assessed using the Hearing Handicap Inventory[48] and assessed symptoms at the time of clinical evaluation. For each item, participants were asked to report the age (in years) at first occurrence. If onset of symptoms was prior to age of germ cell tumor diagnosis, those responses were not considered when assigning severity grade.
** [h] A total of 1,321 patients underwent audiometric assessment, among whom 1,202 received one of the chemotherapy regimens shown in this table. Hearing loss was defined following methods in Frisina et al.[13] using American Speech-Language-Hearing Association criteria.[17]
†† [i] Creatinine measurements were available for the first 976 enrolled TCS, of whom 369 received BEPX3, 299 received EPX4, 181 received BEPX4, 27 received VIPX4/VIPX5, and 14 received cisplatin-based chemotherapy ≥5 cycles. The estimated glomerular filtration rate was calculated following methods in Levy et al.[18] See Table A1 for details.

Supplementary Table 4. Comparison* of Unemployment Status Between Testicular Cancer Survivors in the Platinum Study and a Population from the Behavioral Risk Factor Surveillance System (BRFSS) Which Includes Other Cancer Survivors

	**Platinum Study***		**Normative population (BRFSS)†**	
**Age at assessment**	**Time (years) since chemotherapy completion, median [range]**	**Unemployed N (%)**	**Unemployed % (95% CI)**	**P‡**
18-24	1.4 [1.1, 3.8]	19 (20.2%)	2.2% (1.7, 2.7)	<.001‖
25-29	2.1 [1.0, 9.1]	15 (6.8%)	1.9% (1.3, 2.5)	<.001‖
30-34	1.4 [1.0, 6.7]	17 (7.0%)	1.9% (1.4, 2.4)	<.001‖
35-39	3.9 [1.0, 20.1]	9 (3.7%)	2% (1.5, 2.5)	<.001‖
40-44	2.8 [1.1, 4.8]	5 (2.4%)	2.3% (1.8, 2.8)	.05
45-49	3.5 [1.4, 20.1]	7 (4.2%)	2.5% (1.7, 3.2)	<.001‖
50-54	3.5 [1.1, 14.7]	10 (7.3%)	3.2% (2.5, 3.8)	<.001‖
55-59§	6.5 [5.1, 14.9]	3 (3.7%)	3.2% (2.7, 3.7)	.10

* Restricted to non-Hispanic white males responding ‘Unemployed’ when asked about current employment status.
† Restricted to non-Hispanic white males from the Centers for Disease Control and Prevention BRFSS responding ‘out of work for 1 year or more’ when asked about current employment status (https://nccd.cdc.gov/weat/index.html#/crossTabulation/view). BRFSS patients from Guam, the Virgin Islands, and Puerto Rico were excluded. In contrast to the main Table 4, Table A4 also includes BRFSS patients with a history of cancer. The results are not materially different.
‡ P values are from the Rao-Scott adjusted Chi-square test, using the SAS SURVEYFREQ procedure with the BRFSS sampling weights and a sampling weight of 1 for the Platinum study.
§ No TCS over the age of 59 reported being unemployed. Among the BRFSS normative population, 2.5% (95%: CI 2.0 to 2.9) age 60-64 and 0.7% (95% CI: 0.5 to 0.8) age 65+ reported being unemployed.
‖ Significant after controlling for multiple testing false discovery rate. False discovery rate alpha set to 0.05.
